# The Janus Face of Follicular T Helper Cells in Chronic Viral Infections

**DOI:** 10.3389/fimmu.2018.01162

**Published:** 2018-05-25

**Authors:** Ute Greczmiel, Annette Oxenius

**Affiliations:** Institute of Microbiology, ETH Zürich, Zürich, Switzerland

**Keywords:** follicular helper cells, chronic viral infection, antibody responses, germinal center, viral evolution

## Abstract

Chronic infections with non-cytopathic viruses constitutively expose virus-specific adaptive immune cells to cognate antigen, requiring their numeric and functional adaptation. Virus-specific CD8 T cells are compromised by various means in their effector functions, collectively termed T cell exhaustion. Alike CD8 T cells, virus-specific CD4 Th1 cell responses are gradually downregulated but instead, follicular T helper (T_FH_) cell differentiation and maintenance is strongly promoted during chronic infection. Thereby, the immune system promotes antibody responses, which bear less immune-pathological risk compared to cytotoxic and pro-inflammatory T cell responses. This emphasis on T_FH_ cells contributes to tolerance of the chronic infection and is pivotal for the continued maturation and adaptation of the antibody response, leading eventually to the emergence of virus-neutralizing antibodies, which possess the potential to control the established chronic infection. However, sustained high levels of T_FH_ cells can also result in a less stringent B cell selection process in active germinal center reactions, leading to the activation of virus-unspecific B cells, including self-reactive B cells, and to hypergammaglobulinemia. This dispersal of B cell help comes at the expense of a stringently selected virus-specific antibody response, thereby contributing to its delayed maturation. Here, we discuss these opposing facets of T_FH_ cells in chronic viral infections.

## Introduction

Non-or poorly cytopathic viruses like human immunodeficiency virus 1 (HIV-1), hepatitis B virus (HBV), and hepatitis C virus (HCV) in humans or lymphocytic choriomeningitis virus (LCMV) in mice can induce persistent infections employing several mechanisms to evade control by the immune system. Continuous high-level viral replication and therefore high viral burden in the host is a major factor leading to numeric reduction and functional impairment of virus-specific cytotoxic CD8 T cells and Th1 CD4 T cells, collectively termed T cell exhaustion [reviewed in Ref. (1–3)]. In this setting, immune effector functions being less prone to induce immunopathology, like the humoral arm of immunity, are beneficial to contain viral spread (4–10). Especially, virus-neutralizing antibodies can inhibit new infection of host cells and thereby effectively limit viral spread. However, isotype-switched neutralizing antibodies often appear very late after the onset of persistent viral infections, being frequently delayed for several weeks to months (5, 11, 12). B cell dysregulation, including hypergammaglobulinemia and polyclonal B cell activation, contributes to the late emergence of virus-neutralizing antibodies (13, 14). Furthermore, mutational viral evolution results in selection of variants that escape the neutralizing antibody response, promoting persistence of the infection (12, 15–22).

Isotype-switched antibody responses are elicited in a T-help-dependent manner, being regulated by the interaction between follicular T helper (T_FH_) cells and cognate B cells (23). Activity of T_FH_ cells is regulated by the transcriptional repressor B cell lymphoma (Bcl)-6 (24–26) which sustains, among other functions, upregulation of the chemokine receptor CX-chemokine receptor (CXCR) 5 that in turn mediates localization of T_FH_ cells to the B cell follicle (27–29). There, T_FH_ cells initiate B cell differentiation into either short-lived plasmablasts or germinal center (GC) B cells (30–33). Conversely, contact between T_FH_ cells and cognate B cells is necessary to induce differentiation of T_FH_ cells into GC T_FH_ cells and to sustain their T_FH_ phenotype (34–37), albeit this is disputed to also hold in case of persistent viral infections (38). T_FH_ cells mediate affinity selection of B cells that have undergone proliferation and somatic hypermutation (SHM) by delivering survival signals *via* ICOS, CD40 ligand (CD40L), and the cytokine IL-21, depending on the affinity of the B cell for a given antigen (39–41). Therefore, T_FH_ cells are essential for the induction and maintenance of the GC response.

**Figure 1:**
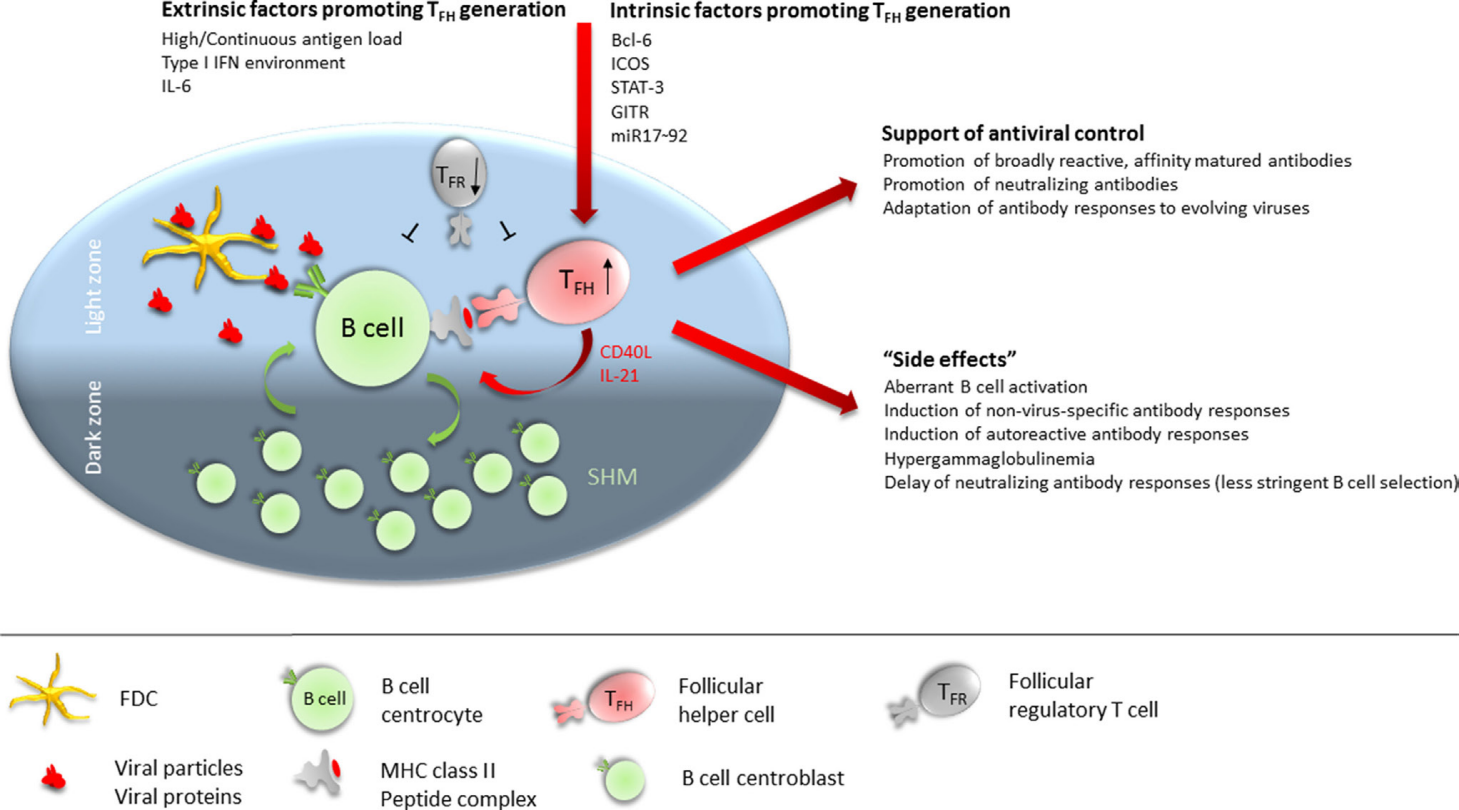
Follicular T helper (T_FH_) cells at the cross-road of helping versus inhibiting. T_FH_ numbers are numerically increased in many chronic viral infections. Extrinsic factors contributing to promote T_FH_ differentiation during chronic viral infections include continuous high antigen load, sustained type 1 IFN environment, and IL-6 availability. Intrinsically, Bcl-6, ICOS, signal transducer and activator of transcription (STAT)-3, GITR, and miR17–92 expression in CD4 T cells is required for (efficient) T_FH_ differentiation. In the germinal center (GC), T_FH_ cells preferentially localize to the light zone (LZ) where they interact *via* their TCR with B cells presenting antigenic peptides on MHC class II. B cells acquire antigen from follicular dendritic cells (FDCs) in the LZ which serve as antigen depot. FDCs retain antigen in form of antibody–antigen complexes or opsonized antigen *via* Fc and complement receptors. Cognate interaction between B cells and T_FH_ provides survival, proliferation, and differentiation signals to the B cell in form of CD40 engagement and IL-21 supply. B cells will then either differentiate into antibody-secreting plasmablasts and long-lived plasma cells, into memory B cells, or enter the GC dark zone where the proliferate and undergo somatic hypermutation of their antibody variable regions before re-entering the LZ for selection of high-affinity B cells clones. Sustained activity of T_FH_ cells is required throughout chronic viral infection to promote broadly reactive, affinity matured, and neutralizing antibodies and to adapt antibody specificity to emerging viral variants. Conversely, the high numbers of T_FH_ cells present during many chronic viral infections render the GC LZ B cell activation and selection process less stringent, leading to aberrant B cell activation, induction of non virus-specific antibodies (including autoantibodies), hypergammaglobulinemia, and delayed generation of neutralizing antibody responses. Further contributing to a dysregulated T_FH_/B cell interaction in GCs is a dysbalanced ratio of T_FH_:T_FR_ cells, often with reduced numbers of follicular regulatory T (T_FR_) cells in chronic viral infections.

Interestingly, T_FH_ cells accumulate during the persistent phase of viral infections with non- or poorly cytopathic viruses (8, 38, 42, 43) while differentiation of naïve CD4 T cells into Th1 CD4 T cells is largely abrogated in this phase due to a sustained IFN-I environment (44). The expansion of the T_FH_ population is most likely driven by follicular dendritic cell (FDC)-derived IL-6 signaling *via* signal transducer and activator of transcription (STAT)-3 (8, 43, 45), and the prolonged persistence of viral antigen in the host environment (46). It would be intriguing to conjecture an essential role of the sustained expansion of the T_FH_ cell population for the eventual induction of the virus-neutralizing antibody response and also adaptation of the protective response to an evolving virus. However, accumulation of T_FH_ cells might also contribute to the observed B cell dysregulation and thereby delay of the neutralizing antibody response (Figure 1). Here, we discuss evidence for both, promotion of late emergence of virus-neutralizing antibodies and dysregulated B cell responses in the context of chronic viral infections, focusing on experimental LCMV infection in mice and HIV-1, HCV, and HBV infection in humans (Table 1).

**Table 1:** Regulation and role of T_FH_ cells in chronic viral infections.

Follicular T helper (T_FH_) cells	Impact	Consequence	Reference	
**Lymphocytic choriomeningitis virus (LCMV) infection**				
Sustained T_FH_ activity	Generation of LCMV-neutralizing antibodies	Eventual control of infection	(7)	Positive role of T_FH_ for antibody responses and eventual virus control
Lack of T_FH_ cells from the onset of infection	Impaired antibody response	Sustained persistent infection	(8, 10, 38, 86–88)	
Increase of T_FH_ cells by NK cell depletion	Improved antibody response	Enhanced/accelerated virus control	(89)	

Sustained high levels of T cell help	Polyclonal B cell activation (including autoreactive B cells)	Hypergammaglobulinemia	(13, 202)	Negative impact of sustained T_FH_ responses

**Simian immunodeficiency virus (SIV)/HIV/hepatitis C virus (HCV)/hepatitis B virus (HBV) infection**				
High frequencies of T_FH_ cells in SIV infection	Correlation with high-affinity SIV-specific antibodies		(43, 90)	Positive role of T_FH_ for antibody responses
Reduction of follicular regulatory T (T_FR_) cells in SIV infection	Increased T_FH_ cell numbers	High avidity antibodies to SIV gp120	(96, 126)	
High frequencies of (functional) (c)T_FH_ cells in HIV, HCV, and HBV infection	Broad (neutralizing) antibody responses	Positive correlation with antibody affinity	(42, 91–95, 97, 98)	
T_FH_ in HCV infection	Reduced IL-21 production	Normal *in vitro* help to B cells	(124)

Reduced T_FH_ frequencies in spleen in SIV infection	Reduced SIV-specific IgG titers		(107)	
Loss of T_FH_ cells in advanced chronic SIV infection	Impairment of B cell response	Progression to AIDS	(100)	Negative impact of sustained T_FH_
cT_FH_ with impaired function in HIV infection	No correlation with neutralizing antibodies	Impaired function	(95, 99)	
Reduced T_FH_ function in HIV infection	Caused by PD ligand 1 (PD-L1) expression on B cells	Blockade of PD-L1 restores T_FH_ function	(123)	
Increased T_FR_ response in SIV and HIV infection	Insufficient germinal center response		(127)	
Increased T_reg_ and B_reg_ in HCV and HBV infection	Impaired antibody response?		(108, 130–132)	
Direct infection of T_FH_ by HIV/SIV	Impaired function	Viral reservoirs	(135–137)	
Sustained high levels of T cell help in SIV, HIV, HCV, and HBV infection	Polyclonal B cell activation s (including autoreactive B cells)	Hypergammaglobulinemia	(42, 43, 199, 200, 203–205)	

*Green: positive role of TFH cells in chronic infections*. *Orange: negative role of TFH cells in chronic infections*. *White: unassigned role of TFH cells in chronic infection*.

## T_FH_ Cells

Follicular T helper cells are the main regulators of T-help-dependent antibody responses (23). Instruction of T_FH_ cell differentiation is mediated in two steps. Priming of CD4 T cells that commit to the T_FH_ cell lineage takes place in the T cell zone and is mediated by conventional DCs or monocyte-derived DCs (47, 48). In a second step, differentiation to T_FH_ cells is further instructed and the T_FH_ phenotype stably established by interactions between primed T_FH_ cells and B cells at the border between T cell and B cell zone.

Which factors/cytokines instruct T_FH_ differentiation is not entirely resolved, but both IL-6 and IL-21 can induce T_FH_ differentiation *via* signaling through the transcription factor STAT-3 (49, 50). In the context of a persistent LCMV infection, it has furthermore been shown that late FDC-derived IL-6 is essential for T_FH_ cell maintenance and eventual control of the infection (8).

CD4 T cells differentiating to T_FH_ cells upregulate the hallmark transcriptional repressor Bcl-6 (24–26). Bcl-6 promotes commitment to the T_FH_ cell lineage by repression of Blimp-1, which mediates expression of genes that are involved in the differentiation into other CD4 T cell lineages (24). Furthermore, Bcl-6 promotes localization of T_FH_ cells toward the B cell follicle where T-help-dependent antibody responses take place. This is achieved in two different ways. For one, Bcl-6 represses the expression of molecules promoting localization in the T cell zone or egress from secondary lymphoid organs, i.e., CC chemokine receptor-7, Epstein–Barr virus-induced G-protein-coupled receptor (EBI)-2, or P-selectin glycoprotein-1 (51–53). Furthermore, Bcl-6 stabilizes the expression of CXCR5 on T_FH_ cells, which is upregulated by the transcription factor achaete-scute homolog-2 (ASCL-2) upon priming (54). CXCR5 is essential for the localization of T_FH_ cells toward the CXCL13-rich B cell follicles (27–29). T_FH_ cells can further be distinguished by expression of other typical markers, which have important functions in mediating cognate interactions with B cells and thus sustaining antibody responses. Among these markers are the costimulatory molecules inducible T-cell costimulator (ICOS) and CD40L, the immunoregulatory molecule PD-1, their hallmark cytokine IL-21, and the T cell adaptor protein SAP (23, 55–58). Expression of these markers is moderate after priming and needs to be sustained and increased by interaction of T_FH_ cells with cognate B cells and by ICOS signals delivered by ICOS ligand expressing bystander B cells in the interfollicular zone (34–37). These first interactions between T_FH_ cells and B cells also determine the differentiation of T_FH_ cells into GC T_FH_ cells that induce and maintain the GC response and play an important role in the positive selection of affinity matured B cell clones. Expression of T_FH_ markers is highest in GC T_FH_ cells (37, 59, 60). After an immune response, some T_FH_ cells have been shown to differentiate into long-lived memory cells, which downregulate some of their typical T_FH_ markers like CXCR5, Bcl-6, and PD-1 (61, 62).

In humans, there have been further reports about circulating T_FH_-like cells that express CXCR5 and display a memory phenotype. Their expression of ICOS, PD-1, and Bcl-6 is reduced as well. However, these cells are efficient producers of IL-21 and IL-10 in *in vitro* coculture and effective inducers of B cell differentiation (23).

Closely related to T_FH_ cells and equally important for the regulation of the GC responses are the so-called follicular regulatory T (T_FR_) cells. These are derived from thymus-derived T regulatory (T_reg_) cells, which adopt some T_FH_ cell characteristics, like CXCR5 and Bcl-6 expression, to be able to migrate into B cell follicles. However, T_FR_ cells lack expression of CD40L, IL-4, or IL-21 and have a higher expression of PD-1 and negatively regulate the GC response (63–65).

## GC Response

The first encounter between T_FH_ cells and activated B cells occurs in the interfollicular zone which lies at the border between T cell zone and B cell follicle (66–68). Here, interaction between T_FH_ cells and cognate B cells induces a first round of B cell proliferation and instructs them to undergo one of three possible differentiation pathways. Either B cells undergo differentiation into short-lived extra-follicular plasmablasts, which produce a first wave of low affinity antibodies, or into GC-independent memory B cells or into GC B cells (30–33, 69). B cells destined to induce the GC response migrate with a subset of T_FH_ cells, GC T_FH_ cells, further into the B cell follicle. This migration is mediated by downregulation of EBI2 and upregulation of Sphingosine-1-phosphate receptor 2 on both B and T_FH_ cells (66, 69–71).

The GC is partitioned into two distinct zones, the dark zone (DZ) and the light zone (LZ). In the DZ the cytokine CXCL12 is predominantly produced while the cytokine CXCL13 is predominantly produced in the LZ. Thereby, localization of B cells in DZ and LZ is controlled by differential expression of the chemokine receptors CXCR4 (migration into DZ) and CXCR5 (migration into LZ) (72). In the DZ, B cells undergo sequential rounds of proliferation (73–76). During this process, B cells upregulate the activation-induced cytidine deaminase (AID) which introduces point mutations into the variable regions of the B cell expressed BCRs/antibodies, a process termed SHM (77–79). Thereby, clonal B cell variants with different affinities toward one given antigen are generated. The activity of AID is also essential for class-switch reactions, which change the isotype of the antibodies (77, 79).

As SHM is a random process, it is necessary for B cells to undergo a selection process to ensure affinity maturation of the antibody repertoire and to exclude B cells that lost affinity for one antigen, decreased their affinity or even developed into autoreactive B cells. This selection process takes place in the LZ of the GC where also most of the GC T_FH_ cells and FDCs are located (73, 75, 76). Upon entry into the LZ, B cells take up antigen which is stored on/presented by FDCs *via* their mutated BCR according to their affinity toward the antigen. Afterward, B cells present processed antigen to cognate GC T_FH_ cells *via* their surface MHC II molecules. Higher affinity B cells are believed to have a competitive advantage in taking up FDC-stored antigen and thus are able to present more antigen on their surface MHC II molecules (80). The amount of presented antigen determines the amount of survival signals *via* ICOS, CD40L, and IL-21 a B cell clone receives from cognate GC T_FH_ cells (39, 40, 76).

B cell clones that do not receive sufficient survival signals and therefore are negatively selected undergo apoptosis mediated by binding of Fas, expressed by the B cell, to FasL, expressed by the GC T_FH_ cell (41, 81). Positively selected B cell clones either undergo another round of proliferation and SHM in the DZ or leave the GC reaction as long-lived plasma cells or memory B cells (74, 75, 82). B cells with the highest affinity may preferentially differentiate into plasma cells (69, 83, 84). B cell clones with a lower affinity, however, rather differentiate into memory B cells (82).

## T_FH_ Cells are Essential for the Emergence of Virus-Neutralizing Antibodies and Control of Persistent Viral Infection

The role of T_FH_ cells for viral control during persistent viral infections, which is assumed to be dependent on development of neutralizing antibodies during the GC response (13, 16, 85), has been widely studied in the setting of persistent LCMV infection. For example, mice harboring a constitutive CXCR5 deficiency, and therefore being unable to develop T_FH_ cells (and B cell follicles), exhibit an abrogated antibody response and prolonged viral persistence (38). Likewise, IL-6^−/−^ (8), IL-6 signaling-deficient (10), STAT3^−/−^ (86), glucocorticoid-induced tumor necrosis factor receptor related protein (GITR)-deficient mice (87), and mice with a T cell-specific deletion of the miR17–92 family of microRNAs (88) fail to elicit or maintain a T_FH_ cell response upon (persistent) LCMV infection and are unable to eventually control the infection. Conversely, increasing the number of T_FH_ cells by NK cell depletion accelerated viral clearance by improving the virus-specific antibody response (89).

Similar correlations between T_FH_ cells and the appearance of protective antibody responses were observed in other persistent viral infections, e.g., with simian immunodeficiency virus (SIV), where the frequency of T_FH_ cells positively correlated with the appearance of high-affinity SIV-specific antibodies in infected rhesus macaques (RM) (43). These T_FH_ cells adopted a Th1-like profile regarding their chemokine receptor and cytokine expression (90). Furthermore, the quantity of T_FH_ cells was higher in slow/non-progressor SIV-infected RMs, in which the virus was better contained, as compared with progressor SIV-infected RMs. The increase in T_FH_ cell numbers in slow progressor correlated with higher titers of SIV-specific IgG antibodies in serum of infected RMs (43). Also, in chronically infected HIV-1, HCV and HBV patients, increased frequencies of a circulating population of cells with T_FH_ characteristics (cT_FH_) (CXCR5^+^CXCR3^−^PD-1^+^) were observed (42, 91–98), which seemed closely related to GC T_FH_ cells, based on their gene expression and cytokine profile. These were able to induce B cell differentiation *in vitro* and correlated with the appearance of broadly HIV-neutralizing antibodies (91, 92). In HIV controllers, an expanded population of functional gp120-specific T_FH_ cells in blood correlated with gp120-specific B cell frequencies (93). However, other studies reported a reduced capacity of cT_FH_ cells to provide help to B cells in (advanced) HIV-1 infected individuals (95, 99) or even loss of T_FH_ cells in SIV-infected RMs (100).

Another indication implicating T_FH_ cells in the eventual emergence of virus-neutralizing antibodies during persistent viral infection is the high frequency of somatic mutations in the variable regions of these antibodies (11, 19, 101, 102). SHM predominantly takes place in the GC and selection of high-affinity clones is supported by GC T_FH_ cells (76). It is therefore tempting to speculate that continued activity of T_FH_ cells during chronic viral infections is required for a continuous selection process of virus-specific B cell clones. This results not only in a continuous increase of their affinity toward viral antigens but also allows them to evolve to bind (and neutralize) to viral quasi species that emerge *in vivo* under selection pressures.

Indeed, we recently presented experimental evidence that sustained presence of CXCR5^+/+^ or Bcl6^+/+^ T_FH_ cells is strictly required for the (late) emergence of LCMV-neutralizing antibodies. Using a novel *in vivo* experimental system allowed conditional depletion of specifically T_FH_ cells or all LCMV-specific CD4 T cells during established persistent LCMV infection, after the initial establishment of the virus-specific IgG antibody response (7). This permitted, in contrast to previous studies (8, 10, 38, 86), to examine the function of T_FH_ cells and LCMV-specific CD4 T cells during persistent viral infection beyond the mere induction of the virus-specific antibody response. This study revealed that LCMV-specific T_FH_ cells (i.e., CXCR5^+/+^ or Bcl6^+/+^ CD4 T cells) were dispensable for maintaining overall LCMV-specific IgG titers and LCMV-specific IgG secreting plasma cells in spleen and bone marrow. By contrast, continued presence of LCMV-specific CD4 T cells was required to maintain overall LCMV-specific IgG titers as well as LCMV-specific IgG secreting plasma cells in bone marrow, suggesting that non-T_FH_ LCMV-specific CD4 T cells are able to support an extra-follicular response to maintain the pool of LCMV-specific antibody-secreting cells and hence LCMV-specific IgG titers. However, sustained activity of T_FH_ cells was strictly required for the development of LCMV-neutralizing antibodies by GC B cells (7), as conditional depletion of T_FH_ cells reduced GC B cell numbers and abrogated emergence of antibodies with neutralizing capacity. Moreover, T_FH_ cells seemed to be essential in driving the adaptation of the IgG response toward the contemporaneous circulating LCMV species, lending support to the notion that sustained T_FH_ activity is important for continued selection of B cells. Importantly, the appearance of neutralizing antibodies was required for eventual control of an established persistent LCMV infection, demonstrating the importance of these antibodies and sustained presence and activity of T_FH_ cells for control of a persistent infection in absence of overt immunopathology (7).

Nevertheless, the belated appearance of neutralizing antibodies in the setting of such persistent infections indicates possible restrictions of T_FH_ cell function and/or their interactions with cognate B cells, which are discussed in the following sections.

## Factors Curtailing T_FH_ Cell Function upon Persistent Viral Infection

Optimal delivery of T_FH_ cell help to cognate B cells as well as optimal T_FH_ cell differentiation includes a chain of distinct steps at specific localizations in lymphoid tissue as well as a series of cell–cell interactions (23). With respect to localization-dependent processes, the structural integrity of secondary lymphoid organs is crucial allowing for initial encounter of activated CD4 and B cells as well as the establishment of GCs in the B cell follicle, including dark and LZ as designated compartments for proliferation, SHM and B cell selection. Cell–cell interactions that support B cell activation and production of (affinity-matured) antibodies comprise direct contact between activated CD4 T cell and cognate B cells initially at the T/B border, and later between T_FH_ cells and cognate B cells in the GC LZ. Interference with any of these steps might lead to suboptimal antibody responses, which negatively affects control of persistent viral infections.

### Destruction of Lymphoid Architecture

One possible influence on the establishment and the quality of T_FH_ and GC B cell responses upon persistent viral infection is (immune-mediated) destruction of the lymphoid tissue architecture (11, 103–110). In chronic LCMV infection, this destruction is largely due to CD8 T cell-mediated cytotoxic activity directed against infected stromal cells as well as sustained type 1 IFN signaling and has been shown to hamper cognate interactions between T and B cells (11, 103–105, 111, 112). In SIV or HIV infection, immune activation-induced fibrosis of lymphoid tissues seems to play a major role in functional deterioration of secondary lymphoid organ structure and function, mediated by T_reg_-dependent transforming growth factor-beta 1 signaling and ensuing collagen deposition (109, 110).

Simian immunodeficiency virus-infected RMs with an expanded T_FH_ cell population and increased SIV-specific antibody responses displayed a more intact lymph node (LN) structure as compared with fast progressing SIV-infected RMs with a less expanded T_FH_ cell compartment (43, 107). This indicates that an intact lymphoid architecture is beneficial for virus-specific antibody responses and containment of the persistent infection.

Although destruction of lymphoid organ architecture is often attributed to cytotoxic CD8 T cells (104), an additional involvement of cytotoxic CD4 T cells during persistent LCMV infection has been shown (113). Cytotoxic CD4 T cells specifically targeted marginal zone (MZ) B cells, MZ macrophages, and metallophilic macrophages (113), subsets which have been implicated in the optimal induction of antibody responses (114–116). Analogous, depletion of MZ B cells was also reported in the context of persistent HIV infection (117) and a strong T helper response, possibly comprising cytotoxic CD4 T cells, is associated with low neutralizing antibody titers in persistent HCV infection (118).

During persistent LCMV infection, restoration of the lymphoid tissue architecture is closely associated with the onset of the neutralizing antibody response occurring between d40 and d80 post-infection (pi) (5, 103). During acute LCMV infection, lymphoid architecture is disrupted by day 8 pi and full reorganization, initiated by viral clearance and contraction of the CD8 T cell response, is only completed by d25 pi (103). During persistent LCMV infection, due to persistence of viral antigen and prolonged activity of CD8 and CD4 T cells before undergoing T cell exhaustion (1, 119–121), disruption of the lymphoid organ architecture is likely protracted as compared with acute LCMV infection. This underscores the relevance of lymphoid tissue reorganization and the onset of the LCMV-neutralizing antibody response, further emphasizing the beneficial effect of an intact lymphoid architecture and thereby optimal T and B cell interactions for the development of virus-neutralizing antibodies.

### B Cell Dysfunction

In the context of HIV and SIV infection, B cell dysfunction was reported by a number of studies, characterized by loss of naïve and resting memory B cells, increases of activated B cells and tissue-like memory B cells, expansion of regulatory B cells, and altered functionality [reviewed in Ref. (122)].

In SIV or HIV infection, B cells were reported to actively render T_FH_ cells ineffective in delivering help to B cells. GC B cells isolated from HIV or SIV-infected individuals/animals displayed a higher expression of PD ligand 1 (PD-L1) as compared with B cells isolated from healthy donors. Therefore, T_FH_ cells received more signals *via* PD-1 during HIV/SIV infections, which mediated downregulation of IL-21 and IL-4 expression, and at the same time had a negative impact on T_FH_ cell survival and proliferation (123). This impaired their B helper capacity, as observed in *in vitro* coculture experiments. Blocking of PD-L1 on B cells derived from HIV or SIV-infected donors, however, increased the ability of T_FH_ cells to provide help to B cells as well as their cytokine expression (123). This also proved that T_FH_ cells are in principle capable of providing sufficient help to B cells.

Furthermore, cT_FH_ cells exhibiting reduced IL-21 expression as compared with healthy donors were identified in blood of persistently HCV infected patients (124). Surprisingly, however, in contrast to HIV and SIV infection, these cells proved to be capable of providing help to B cells in *in vitro* coculture experiments (124). These differences might be due to the different usage of B cell subsets in the coculture settings. Cocultures in the context of HIV/SIV infection were set up with GC-enriched B cells (123) while cocultures in the context of HCV infection used memory B cells (124). It is also conceivable that different non-or poorly cytopathic viruses use different mechanisms to render the antibody response ineffective upon persistent infection.

### Altered Ratios of Regulatory Cells

In the setting of a recent HIV vaccination trial, it was established that the ratio of T_FH_ cells to GC B cells is more important for the quality of the antibody response and eventual emergence of neutralizing antibodies than the total cell numbers. In this context, interaction of few GC B cells with one T_FH_ cell was positively correlated with the occurrence of broadly neutralizing antibodies (bnab) (125). Furthermore, GC responses are subject to regulation by regulatory cells, in particular by T_FR_ cells, which control the GC response to prevent aberrant production of antibodies (64). It has been shown in some studies that the frequency of T_FR_ cells is reduced upon persistent infection with HIV and SIV (96, 126), albeit other studies have reported an increase of this population in HIV and SIV infection (127). Decreased levels of T_FR_ cells favor the observed expansion of T_FH_ cells and could indicate a less regulated GC response, hampering the induction of protective antibody responses for instance by a less stringent selection process and promoting unspecific B cell activation leading to hypergammaglobulinemia. Conversely, an expanded T_FR_ population might contribute to inefficient GC responses [reviewed in Ref. (128)].

Also, in the context of persistent LCMV infection of lymphopenic mice lacking regulatory T cells, the induction of protective antibody responses was shown to be impaired (46, 129). Adoptive transfer of T_reg_ improved the LCMV-specific antibody response and viral clearance drastically (129), proving the importance of balanced ratios between regulatory cells and T_FH_ and GC B cells during the GC reaction.

Interestingly, in contrast to SIV, HIV and LCMV infection in lymphopenic mice, persistently HCV- or HBV-infected patients displayed an increase of regulatory B cells and T_regs_ as compared with healthy donors. This was associated with increases in IL-10 expression and increased PD-L1 expression on T_reg_ cells (108, 130–132), which together might impair HCV- and HBV-specific antibody responses being associated with poor virus elimination and damage to lymphoid tissue (108).

### Accumulation of T_FH_ Cells Which Are Not Specific for Antigens Carrying Neutralizing Epitopes

Upon HIV infection, a predominant expansion of T_FH_ cells that are specific for group-specific antigen is reported (42). However, induction of bnab seems to be associated with Env-specific T_FH_ cells (91). Therefore, specific expansion of T_FH_ cell populations, which are not recognizing the protein carrying neutralizing epitopes, could further contribute to the delayed emergence of neutralizing antibodies. T_FH_ cells with other specificities would predominantly favor the survival of B cells expressing antibodies that are not specific for the neutralizing epitope. However, such intramolecular T cell help does not seem to be generally required and depends on the structure of the B cell activating viral antigen. While individual viral proteins engaging specific BCR would require intramolecular help, B cells interacting with intact or defective virions or virus-derived protein complexes could also be activated by T_FH_ cells that are not necessarily specific for the protein containing the neutralizing epitopes (133, 134). Thus, it would be interesting to understand in more detail the structures of the selecting viral antigens/antigen complexes in the context of persistent viral infections to delineate more precisely the specificities of beneficial T_FH_ responses.

### Direct Infection of T_FH_ and T_FR_ Cells in HIV and SIV Infection

Upon HIV and SIV infection, T_FH_ functionality is additionally compromised by their direct infection. CXCR5^+^ CD4 T cells are generally more permissive for HIV and SIV infection as compared with CXCR5^−^ CD4 T cells, with GC T_FH_ exhibiting the highest permissiveness (135–137). T_FR_ cells are also highly permissive for HIV infection—even more so than T_FH_ cells (138).

Surprisingly, infected T_FH_ cells are not directly eliminated as compared with infected CXCR5^−^ CD4 T cells. This might be due to the fact that only few CD8 T cells express CXCR5 and therefore cannot efficiently enter the B cell follicle where the infected T_FH_ cells reside (136, 139, 140). In that way, T_FH_ cells serve as viral reservoirs. However, during SIV and HIV infection, the CXCR5^+^ CD8 T cells that enter the GCs seem to contribute to control of infection (141), or alternatively negatively regulate T and B cells responses *via* IL-10 and Tim3-dependent processes (142). Infected GC T_FH_ cells downregulate T_FH_ markers during active viral replication (135) which might negatively affect their B cell helper functions, rendering the induction of antibody responses less effective.

## Viral Evolution Can Mediate Evasion from the Neutralizing Antibody Response—Arms Race between Virus and the Humoral Immune Response

Besides immunological and secondary lymphoid organ topographical factors that might curtail effective T_FH_ responses and thereby induction of neutralizing antibody responses, viral mutation can contribute to the establishment of persistence by escape from imposed immune pressure such as the humoral immune response. RNA viruses are known to evolve upon infection due to a high mutation rate during viral replication with their non-proofreading RNA-dependent RNA polymerase (or reverse transcriptase) and exist as a so-called quasi species in the infected host (143, 144). These high mutation rates allow the rapid adaptation of RNA viruses to changing environments and selective immune pressures (145). Viruses like HIV, HCV and LCMV take advantage of this viral evolution for the establishment of persistence, e.g., by sequential evasion from the adaptive immune response.

In persistent LCMV infection, especially in settings of reduced or absent CD8 T cell responses, escape variants from the neutralizing antibody response emerge that promote persistence of LCMV (15, 16, 146). This escape was mediated by only few amino acid substitutions in the neutralizing epitope contained in GP1 (15). However, LCMV generally has a rather low mutation rate, with 2.6 × 10^−4^ to 5.5 × 10^−5^ mutations per round of replication (147), compared with other RNA viruses with 10^−3^ to 10^−5^ miss-incorporations per copied nucleotide (15, 147, 148). Generally, selection of mutations was reduced or lacking in absence of neutralizing antibodies, indicating positive selection of escape viral variants upon antibody-imposed immune pressure (15). As recently published, escape of LCMV from the neutralizing antibody response also occurs in presence of a normal CD8 T cell response, meaning that neutralization of contemporaneous virus isolates lagged behind neutralization of the inoculating virus (7). This raises the question of how viral diversity is affected in absence of T_FH_ cells. As animals with a conditional depletion of CXCR5^+/+^ T_FH_ cells did not develop effective neutralizing antibodies against neither the inoculum or contemporaneous virus isolates, circulating antibodies most likely exhibited reduced immune pressure on the neutralizing epitopes (7). One would speculate that viral diversity is more restricted in absence of T_FH_ cells as compared with control situations. Whether this prediction holds will have to be investigated in future studies.

Escape from the neutralizing antibody response and subsequent adaptation of the humoral immune response to new viral variants is more extensively investigated in persistent viral infection with HIV or HCV as compared with persistent LCMV infection. HIV and HCV infection share the common feature that the neutralizing antibody response is at first only directed against the autologous virus, while neutralization of heterologous viral variants by bnab is rather rare and only occurs later during infection (22, 149–152). Moreover, in HIV and HCV infection, the neutralizing antibody response toward the autologous virus usually lags behind the concurrent evolution of the viral quasi species, meaning that antibodies isolated from a given time point generally fail to neutralize contemporaneous virus isolates but are able to neutralize isolates from prior time points (22, 149, 152–154). Thus, during persistent viral infections a molecular arms race is taking place between the virus and the humoral immune response.

Mutations conferring escape are mostly accumulating in variable regions of the viral envelope (env), against which neutralizing antibodies are directed, e.g., the variable loops of HIV gp120 (19, 155) or the hypervariable region (HVR) of HCV (156, 157). Either these variable regions cover more conserved neutralizing epitopes or these regions contain the first neutralizing epitopes as in case of the HVR of HCV (20, 155–157). In addition, shielding of neutralizing epitopes by establishment of a glycan shield *via* mutational introduction of glycosylation sites is used by persistently infecting viruses to hamper binding of neutralizing antibodies by steric hindrance (22, 154, 158–162). Generally, neutralizing antibodies detect deglycosylated forms of the virion better than the glycosylated form as shown for HIV or Arenavirus infections (161, 163–165). Glycans reduce the on-rate of the neutralizing antibody and thereby limit their neutralizing capacity (161). In case of HIV infection, some glycans also increase the flexibility of the variable loops of the envelope protein, thereby increasing the binding entropy for neutralizing antibodies, which is unfavorable (166). Interestingly, however, in some HIV-infected patients, neutralizing antibodies that are able to penetrate the glycan shield by binding one or multiple conserved glycans (e.g., glycans at position N332 or N301) and simultaneously to gp120 protein residues (167–171) were elicited. This clearly shows that the humoral immune response is in principle able to develop antibodies that are able to bypass mechanisms conferring escape from the neutralizing antibody response.

Yet, in persistent HIV or HCV infections such bnab that bind to more conserved epitopes like glycan patches occur rather seldom (149, 150, 172–175). Most bnab are characterized by a high amount of somatic mutations, long CDRH3 regions and preferential usage of specific heavy and light chains (175–180). A high rate of somatic mutations can be observed in neutralizing antibodies against Arenaviruses like LCMV as well (11). Precursors of bnabs can be identified already early during the virus-specific humoral immune response upon persistent HIV infection (171, 181–186). The slow development of such precursors toward a bnab, together with the high quantity of somatic mutations, indicates that neutralizing antibodies mature over a prolonged period of time in the GC, including selection by T_FH_ cells to develop the necessary neutralization breadth. Factors curtailing the GC response as described earlier might well contribute to the impaired or delayed emergence of such bnab. Furthermore, to allow the continued development of bnab in the GC response, their evolution/selection has to occur against viral variants that do not undergo complete viral escape from these bnab precursors (187).

Interestingly, diversity of the viral variants and the viral load influence the development of bnabs in HIV infection. Prolonged viremia and a higher diversity of the env are positively correlated with their induction (172, 173, 188–192). However, it is still a matter of discussion whether early diversity of the env (189, 190, 192), as, for example, achieved by superinfections (192) or a high diversity of the contemporaneous env genes is correlated with the emergence of bnab (193). Nevertheless, diversification of the viral variants is often observed before the onset of bnab responses (183, 184). Analogous, protracted viremia in persistent infections with Arenaviruses like LCMV is favorable for the induction of the neutralizing antibody response (11).

So far, little is known about the overall evolution of the LCMV-specific antibody response over the course of a chronic infection. Sustained T_FH_ activity is crucial for the eventual emergence of neutralizing antibodies (7). However, how this sustained T_FH_ activity supports the emergence of neutralizing antibodies is unclear. It could either be *via* continuous rounds of SHM and selection of B cells which would eventually give rise to antibodies with neutralizing capacity. Alternatively, T_FH_ cells might be required for continued recruitment of new B cell clones into the GC response, thereby contributing to an overall broadening of the antibody repertoire. Interestingly, a recent vaccination study in humans repetitively exposed to the malaria parasite *Plasmodium falciparum* revealed that selection of potent B cell precursors from the naïve or memory pool contributed more efficiently to a potent antibody response to a complex antigen than the process of affinity maturation (194).

In the context of a chronic viral infection, it would be interesting to elucidate how viral diversity is reflected/presented in the GC response, leading to selection of the precursors of B cells producing neutralizing antibodies. Generally, GC B cells are dependent on taking up antigen from FDCs for affinity selection (76). However, whether this holds true in a setting with abundant free viral antigen during persistent viral infection still has to be determined. Interestingly, however, FDCs have been shown to be archives of viral quasi species upon HIV infection (195, 196), which would indicate constant binding of viral variants and their presentation. This would suggest that FDCs could also present the newest contemporaneous viral variants to B cells, which are then selected according to their affinity toward these variants. Yet, it remains unclear how fast the turnover rate of antigen presented by FDCs is in the setting of a chronic viral infection, which, in case of slow turnover, might lead to delays in the selection of B cells against the newest contemporaneous viral variants.

Moreover, emergence of bnab upon persistent HIV infection is also determined by the rate at which somatic mutations are acquired by B cell clones. For some bnab families it has been determined that the mutation rate was faster than that of the virus (181, 197), which enabled the host to “overtake” the viral evolution and develop an effective neutralizing antibody response. Concerning the role of T_FH_ cells in the selection process of B cells producing such neutralizing antibodies, it has been established recently that the interaction intensity between T_FH_ cells and GC B cells determines the quantity of proliferation rounds and therefore the quantity of somatic mutations a B cell can acquire (198). Therefore, it would be of interest to determine the influence of T_FH_ cells on the mutation rate of such B cell clones. This could be achieved using the novel *in vivo* experimental model that allows conditional depletion of T_FH_ cells upon persistent LCMV infection (7). Virus-specific plasma cells, developed in presence or absence of T_FH_ cells, could be isolated at different time points pi, and the quantity of somatic mutations in the variable regions of heavy and light chains could be determined by NGS. Isolating contemporaneous virus isolates at the same time point and determining the sequences of their neutralizing epitopes by NGS could be used to relate the evolution kinetics of virus-specific B cells to the evolution of the virus. This approach could also be employed to determine whether the observed preferential usage of specific heavy and light chains by neutralizing antibodies is influenced by the absence of T_FH_ cells. In absence of continuous T_FH_ activity, one could conjecture that the overall diversity of B cell clones is increased, as the selection process is most likely much less stringent in absence of T_FH_ cells, and the overall frequency of somatic mutations in B cells might be reduced due to insufficient selection and consecutive rounds of SHM.

## Does Accumulation of T_FH_ Cells Contribute to Dysregulated B Cell Responses upon Persistent Viral Infection?

During persistent viral infections with LCMV, HIV, SIV, or HCV, dysregulated B cell responses are observed. This includes the induction of hypergammaglobulinemia and polyclonal B cell activation, resulting in the emergence of seemingly virus-unspecific antibodies and in some cases even autoimmune reactive antibodies (199–205). However, in a recent study examining antibody responses toward *Salmonella Typhimurium* infection, it was shown that the seemingly predominantly *Salmonella*-unspecific antibody response was in fact of very low affinity toward *Salmonella* that increased due to affinity maturation in extra-follicular patches (206). Therefore, it would be interesting to investigate whether unspecific antibody responses elicited upon persistent viral infections might also display very low (undetectable in commonly used read-outs) affinities for the virus, which might improve upon affinity maturation and then allows recruitment into the virus-specific antibody response.

The described B cell dysfunctions have been further linked to the delayed appearance of neutralizing antibodies and in the context of persistent LCMV infection have been shown to be dependent on CD4 T cell help to cognate B cells *via* CD40:CD40L signals (13, 202, 207). It is believed that the virus-unspecific B cells acquire viral antigen from the environment and present it *via* their surface MHC II molecules to cognate CD4 T cells. How exactly virus-unspecific B cells acquire viral antigen to present to CD4 T cells and whether they might require signals *via* their BCR to become activated is not fully elucidated. In the setting of persistent LCMV infection, uptake of antigen by LCMV-unspecific B cells is independent of complement receptors (CRs) and FcγR, as knockout mice still display hypergammaglobulinemia (202). A recent study showed in the setting of an acute disseminated encephalomyelitis model with influenza infection, that uptake of the self-antigen myelin oligodendrocyte glycoprotein (MOG) *via* the BCR could occur concurrent with influenza hemagglutinin (HA). This led to the simultaneous presentation of MOG and HA on the MHC II surface molecules of MOG-specific B cells and subsequently their activation *via* HA-specific CD4 T cells (208). This scenario could serve as explanation for the activation of self-reactive B cells in the setting of persistent viral infections and would also indicate participation of BCR signaling pathways. However, this model does not account for virus-unspecific antibody responses toward non-self-antigens, such as for instance against the hapten nitrophenol (202). Another possible pathway that has been proposed to contribute to the uptake of viral antigen by virus-unspecific B cells in the setting of persistent viral infections is pinocytosis (202). Assumingly, due to the high viral burden, the concentration of viral particles and therefore viral antigen would be sufficient to induce sufficient uptake *via* this mechanism from the environment.

Regarding the contribution of T_FH_ cells to dysregulated B cell responses, it has been shown before in settings of autoimmunity that prolonged maintenance of T_FH_ cells, and therefore prolonged maintenance of GC B cells, is one cause for the emergence of autoreactive antibodies (58, 209–213). The selection threshold is lowered in GCs when T_FH_ numbers are increased; thereby permitting the survival of low affinity and self-reactive B cells (214)—a situation that is met during persistent viral infections.

Analogous, in HIV and SIV infection, the expansion of T_FH_ cells observed in LNs of infected individuals correlated with hypergammaglobulinemia and polyclonal B cell activation as well as the deletion of circulating memory B cells (42, 43, 215). Treatment of HIV-infected individuals with antiretroviral therapy reduced T_FH_ cell numbers and at the same time B cell dysfunctions (42, 200), which indicates a connection between expansion of the T_FH_ cell population in persistent HIV and SIV infections and dysregulated B cell responses. Similarly, in persistent HBV infection, the frequency of cT_FH_ cells correlated with the emergence of autoantibodies (205).

## Are there Organ-Specific Differences in T_FH_ Cell Expansion and Function?

It also should be considered when discussing T_FH_ accumulation and its impact on the antibody response that organ-specific differences might exist in specific persistent viral infections. This has been recently addressed in the context of SIV infection (107). Most studies upon persistent SIV and HIV infection have been conducted in blood or LN samples of infected animals/patients. Yet, recently, T_FH_ responses have been analyzed in spleens of SIV-infected RMs (107). In contrast to results obtained from LNs of SIV-infected RMs, the T_FH_ cell frequency in spleen was drastically reduced already in the acute phase of SIV infection as compared with healthy animals. This phenomenon was maintained in the persistent phase of SIV infection. In addition, T_FH_ cells in spleen of SIV-infected RMs expressed less of the T_FH_-associated transcription factors Bcl-6 and c-Maf and instead upregulated transcription factors that counter-regulate T_FH_ cell fate, i.e., Krüppel-like factor-2. This decrease in T_FH_ cell frequency was further associated with reduced titers of SIV-specific IgG antibodies (107). However, T_FH_ frequencies were similar or elevated in LNs of these infected RM as compared with healthy animals and in accordance with previous reports (43, 107, 215). Interestingly, the depletion of T_FH_ cells in the spleen of SIV-infected RMs occurred in the context of severe destruction of the splenic architecture (107). Therefore, it might be possible that differences concerning the preservation of the lymphoid tissue could account for the observed organ-specific differences. Probably, due to the severe destruction of splenic architecture, SIV-infected T_FH_ cells might have enhanced contact with cytotoxic CD8 T cells in the acute phase of infection, which might cause deletion of T_FH_ cells in the spleen. Possibly, also differences in the recruitment of effector cells or different cytokine milieus in the LN and the spleen might influence the maintenance of T_FH_ cells upon SIV infection.

Therefore, organ-specific differences in T_FH_ cell frequency and function have to be taken into consideration as together they might account for the outcome of the virus-specific antibody response.

## Concluding Remarks

Follicular T helper cell function and optimal interactions between T_FH_ cells and cognate B cells often are hampered during persistent viral infections due to several factors. These include sustained increase of T_FH_ cells, leading to non-specific B cell activation and hypergammaglobulinemia at the expense of virus-specific antibodies, destruction of the lymphoid tissue architecture, B cell exhaustion, skewed ratios of regulatory cells to T_FH_/GC B cells or in case of HIV/SIV infection T_FH_ cells being directly infected. Due to these dysregulations, protective virus-specific antibody responses are delayed. Moreover, viruses use different mechanisms to evade recognition by antibodies using, e.g., variable loops or glycan shields to protect neutralizing epitopes. Furthermore, constant viral evolution leads to continued selection of escape variants upon exerted pressure by neutralizing antibodies, which fuels a molecular arms race between virus and the humoral immune response.

Nevertheless, it is clear that sustained activity of T_FH_ cells is essential for the induction of neutralizing, protective antibody responses upon persistent viral infection and that the eventual emergence of these antibodies can afford control of the persistent infection in absence of overt immunopathology.

Therefore, targeting mechanisms that promote optimal T_FH_ cell function and interactions with cognate B cells as well as understanding the underlying mechanisms of the arms race between virus and humoral immune response might serve to improve the induction of neutralizing antibody responses and reduce B cell dysfunctions, thereby improving control of persistent viral infections.

## Author Contributions

All authors listed have made a substantial, direct, and intellectual contribution to the work and approved it for publication.

## Conflict of Interest Statement

The authors declare that the research was conducted in the absence of any commercial or financial relationships that could be construed as a potential conflict of interest.
